# Antifungal Drug Development: Targeting the Fungal Sphingolipid Pathway

**DOI:** 10.3390/jof6030142

**Published:** 2020-08-20

**Authors:** Kyle McEvoy, Tyler G. Normile, Maurizio Del Poeta

**Affiliations:** 1 Department of Microbiology and Immunology, Stony Brook University, Stony Brook, NY 11794, USA; kyle.mcevoy@stonybrook.edu (K.M.); tyler.normile@stonybrook.edu (T.G.N.); 2 Division of Infectious Diseases, School of Medicine, Stony Brook University, Stony Brook, NY 11794, USA; 3 Veterans Administration Medical Center, Northport, NY 11768, USA

**Keywords:** antifungals, fungal infections, drug development, sphingolipids, cryptococcosis, candidiasis, aspergillosis

## Abstract

Fungal infections are becoming more prevalent and problematic due to the continual rise of immune deficient patients as well as the progressive development of drug resistance towards currently available antifungal drugs. There has been a significant increase in the development of antifungal compounds with a similar mechanism of action of current drugs. In contrast, there has been very little progress in developing compounds inhibiting totally new fungal targets or/and fungal pathways. This review focuses on novel compounds recently discovered to target the fungal sphingolipids and their metabolizing enzymes.

## 1. Fungal Infections in Humans and Current Antifungal Drugs

Fungal pathogens are becoming increasingly problematic now more than ever with the rise of immunocompromised individuals, such as patients with HIV/AIDS, those undergoing medical intervention, or those taking immunosuppressant medications [1]. Even though there are approximately 1.5 million fungal species worldwide, only a small subset are pathogenic to humans mainly because most fungi found in the environment cannot grow at the human body temperature of 37 °C. Interestingly, among those species that have adapted to survive and replicate at high body temperature, the sphingolipid pathway and the associated metabolizing enzymes are both highly conserved between humans and these fungi. These are essential for the fungus to cause tissue damage and the consequent disease, however, none of the current clinically available antifungal drugs target the fungal sphingolipid pathway.

Several in depth reviews have been published in recent years that provide information regarding the current standard of care treatment options of clinically available drugs, and therefore we only briefly cover these topics and point the reader to these reports for each brief discussion. Currently, antifungal drugs can be divided in three classes based on their mechanism of action: polyenes, azoles, and echinocandins. (i) Polyenes interact with ergosterol on the fungal membrane and this interaction results in perforations of the fungal cell membrane leading to cell death. Unfortunately, polyenes have also been shown to interact with cholesterol on the host membranes, contributing to the observed cytotoxicity in patients (reviewed in [2]). The most well-known polyene, amphotericin B (AmB), is one of the most potent fungicidal agents on the market with a broad-spectrum of activity, shown to be effective against *Cryptococcus* spp. and most *Candida* spp., but several next generation drugs have been in the making [3,4,5]. (ii) Azoles are a class of antifungal drugs that target lanosterol-14α-demethylase (Erg11), which catalyzes the demethylation of lanosterol to make an important precursor that is eventually converted into ergosterol [6,7]. Several generations of azole drugs have been developed over the years, but fluconazole remains the main azole drug of choice used in the clinic against invasive fungal infections. However, this drug has been accompanied with the inevitable development of fungal resistance by many of the pathogenic species. (iii) Echinocandins are a class of synthetic antifungal compounds that act by inhibiting the synthesis of an essential fungal cell wall component, 1,3-β-d-glucan [8]. Caspofungin, micafungin, and anidulafungin are the main three echinocandin compounds, which cause a myriad of effects dependent on the fungal species. They are fungicidal against most *Candida* spp. and fungistatic against many *Aspergillus* spp. However, when compared to azoles, echinocandins have milder side effects and a better survival rate for the host but only a narrow spectrum of antifungal activity (reviewed in [9]).

Aside from the three main classes mentioned above, flucytosine is an oral drug that indirectly exerts its antifungal effects via molecular mimicry to DNA bases. Flucytosine is a fluorinated analog of cytosine, which becomes incorporated into the fungal cells through cytosine permease. The fluorinated cytosine (5-FC) is a prodrug that is converted into the active antifungal form inside the cell (5-fluorouracil (5-FU)) by fungal cytosine deaminase (Fcy1) [10,11]. 5-FU exerts its antifungal effect through inhibition of protein synthesis in the fungal cells and also inhibits thymidine synthase causing an interference with nucleic acid synthesis, consequently impairing protein synthesis. 5-FU and its derivatives have been shown to cause cytotoxic effects in the host and resistance is growing in certain pathogens including *C. neoformans*.

Due to the limited number of antifungal drugs available, the overuse has led to the increasing development of fungal resistance. Because of this, drug resistance of common fungal pathogens, such as *C. neoformans*, *C. albicans*, and *A. fumigatus*, has become a growing burden on the healthcare system worldwide. Most alarming is the emergence of fungal species, such as *C. auris* and *C. glabrata*, that were already resistant to current antifungal agents [12]. Thus, there is an urgency for the development of novel antifungal drugs with new mechanisms of action to be used alone or in combination with current antifungals. The fungal sphingolipid pathway represents an exciting opportunity to explore an untouched pathway, as many metabolizing enzymes are essential for fungal growth or/and virulence. Additionally, this pathway represents novel targets that are outside of the common targets of clinically available drugs described above.

## 2. The Fungal Sphingolipid Pathway

**Figure 1:**
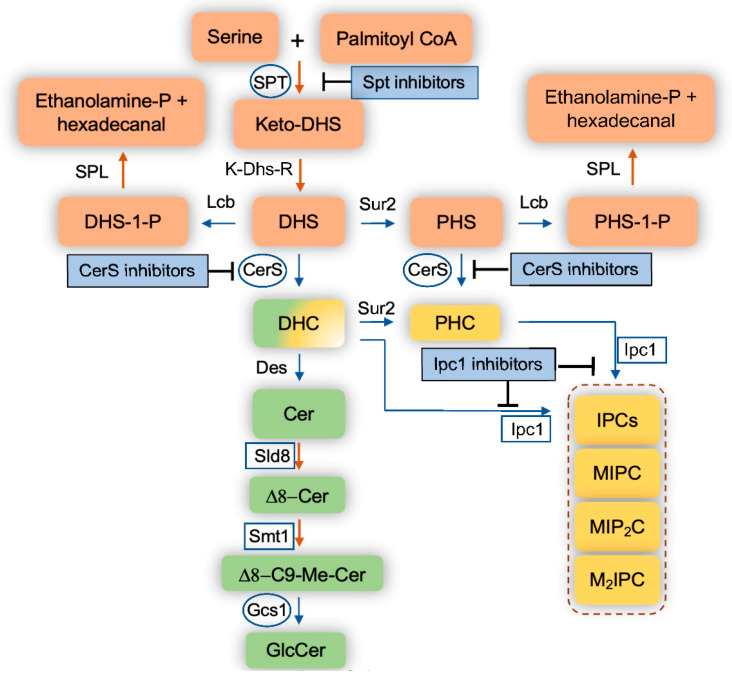
Schematic representation of the sphingolipid pathways in fungi. Blue arrows indicate reversible reactions. Orange arrows indicate irreversible reaction. Squares represent fungal enzymes that have no homologs in mammalian cells. Circles represent fungal enzymes that are significantly different than human homologs. DHS, dihydrosphingosine; PHS, phytosphingosine; DHS-1-P, dihydrosphingosine-1-phosphate; PHS-1-P, phytosphingosine-1-phosphate; DHC, dihydroceramide; PHC, phytoceramide; Cer, ceramide; C9-Me, C9-methyl; GlcCer, glucosylceramide; IPC, Inositol phosphoryl ceramide; MIPC, mannosyl inositol phosphoryl ceramide; MIP_2_C, mannosyl diinositol phosphoryl ceramide; M2IPC, dimannosyl inositol phosphoryl ceramide; Ethanolamine-P; ethanolamine-phosphate; SPT, serine palmitoyl transferase; Lcb, long chain base kinases; K-Dhs-R, keto-dihydrosphingosine reductase; Sur2, sphingolipid hydroxylase; CerS, ceramide synthase; Des, sphingolipid desaturase; Sld8, sphingolipid desaturase 8; Smt1, sphingolipid methyl transferase; Gcs1, glucosylceramide synthase 1; Ipc1, inositol phosphoryl ceramide synthase 1, SPL, sphingolipid lyase.

The fungal eukaryotic cell membrane is a multifaceted cellular site that is made up of several components, most notably sterols, glycoproteins, glycolipids, phospholipids, and sphingolipids [13,14,15]. These components aid in the structural organization of the membranes as well as in the regulation of membrane potential, influx and efflux of metabolites, vesicular transport, and in forming membrane signaling domains known as lipid rafts. Of these components, sphingolipids have gained a lot of attention in the past decade since being uncovered as key components of fungal cellular membranes that participate in essential cellular events. Sphingolipids are complex lipids that have a sphingoid base, such as dihydrosphingosine (DHS), phytosphingosine (PHS), or sphingosine, rather than glycerol as a backbone, to which a fatty acid and various head groups are attached. They are found on both the inner and outer membrane of eukaryotic cells. The major fungal sphingolipids are inositol phosphoryl-ceramides (IPCs) and glucosylceramide (GlcCer), and they are both important for many fungal biological processes including the regulation of fungal virulence [16,17,18,19,20,21,22,23]. IPCs are produced by the action of inositol phosphoryl ceramide synthase 1 (Ipc1) (Figure 1) and it has been shown to be required in *C. neoformans* for intracellular growth within macrophages [21]. GlcCer is produced by GlcCer synthase 1 (Gcs1) (Figure 1) and was shown to be essential in *C. neoformans* growth in a neutral/alkaline (mostly extracellular) environment [23].

During recent years, the enzymes involved in sphingolipid biosynthesis have been explored as potential new targets for the research and development of new antifungal compounds. Interestingly, targeting the function of fungal sphingolipids directly has also been the focus of intense investigations.

Sphingolipid biosynthesis begins with serine and palmitoyl CoA being condensed by serine palmitoyltransferase (SPT) into ketodihydrosphingosine (Figure 1). SPT is encoded by Lcb1, Lcb2, and Tsc3 in *Saccharomyces cerevisiae* and by SPTLC1, SPTLC2, and SPTLC3 in mammalian cells. The ketodihydrosphingosine is then reduced to dihydrosphingosine (also referred to as sphinganine) through NADPH-mediated reduction by a reductase. Afterwards, ceramide synthases add a variety of fatty acids to dihydrosphingosine, producing different species of dihydroceramides. Ceramide synthase is encoded by Lag1, Lac1, and Lip1 in *S. cerevisiae*; Cer1, Cer2, and Cer3 in *C. neoformans*; Lag1 and Lac1 in *C. albicans*; LagA and BarA in *Aspergillus* spp.; and CerS1, CerS2, CerS3, CerS4, CerS5, and CerS6 in mammalian cells. Dihydrosphingosine can also be hydroxylated at position 4 of the sphingosine backbone into phytosphingosine, which can be used as a substrate by ceramide synthases to produce several phytoceramide species. Finally, dihydrosphingosine can be phosphorylated into a bioactive sphingolipid called dihydrosphingosine-1-phosphate by at least two sphingosine kinases (human SK1 (or Sphk1) and SK2 (or Sphk2), and *S. cerevisiae* Lcb4 and Lcb5). These sphingosine kinases can also phosphorylate phytosphingosine (and sphingosine—discussed below) into phytosphingosine-1-phosphate (and sphingosine-1-phosphate).

These phosphorylated sphingolipids are highly soluble and able to traverse in and out of the cell very quickly. The intracellular level of these compounds is extremely low, but it increases 5–10 fold when fungal cells are exposed to high temperatures (e.g., 37 °C) [24], suggesting a key role for sphingolipids in fungal signaling required to protect cells from heat stress. For instance, fungal cells unable to break down these phosphorylated sphingolipids because of the double deletion of the sphingolipid lyase (SPL, Figure 1) (*S. cerevisiae* Dpl1) and the phosphatase (reverse reaction of Lcb4 and Lcb5, in *S. cerevisiae* this phosphatase is called Lcb3), show an approximate 500-fold increase of phytosphingosine-1-phosphate and dihydrosphingosine-1-phosphate levels. As a result, they are able to survive at temperatures upwards of 44 °C, which is 10-fold better compared to wild-type cells [24]. This suggests that the enzymatic activity of the enzymes regulating the intracellular level of these phosphorylated sphingolipids may be highly regulated, particularly when fungal cells move from a low to a high temperature condition. When dealing with environmental pathogenic fungi, such as *Cryptococcus* spp. and *Aspergillus* spp., that are inhaled from the environment (~25 °C) into the lung (~37 °C), these phosphorylated sphingolipid levels may allow the fungus to adapt to the new high temperature environment, thus promoting survival, stimulating fungal growth, and ultimately the development of the fungal disease.

Dihydroceramides, phytoceramides, or ceramides are used to build more complex sphingolipids, such as GlcCers and IPCs. GlcCers are almost exclusively made out of ceramides, as only minor GlcCer species contain dihydroceramides. In contrast to mammalian cells where GlcCer is then used to make very complex sphingolipids, such as ganglio-series, isoglobo-series, lacto-series, and neolacto-series, in fungal cells, GlcCer is the final step and in certain fungi, it represents the pinnacle of the major complex sphingolipids. However, this is not the case in the model yeast *S. cerevisiae*, as this yeast does not produce GlcCer for the lack of Sld8, Smt1, and Gcs1 (Figure 1). Interestingly, fungi making GlcCer have similar chemical structure, which is very different from the structure of mammalian GlcCer. In fungal cells, two additional fungal specific enzymes, Sld8 and Smt1 (Figure 1), modify the sphingosine backbone of the ceramides by adding a double bond in position 8 (Sld8) and a methyl group in position 9 (Smt1). This unique structure of GlcCer gives fungi the ability to replicate at a neutral/alkaline environment [20].

In addition to GlcCer, IPCs are mainly made out of phytoceramides or dihydroceramides, mostly containing very long chain and unsaturated fatty acids, through the action of the inositol phosphoryl ceramide synthase 1 (Ipc1, also called Aur1) enzyme. This is an essential enzyme for fungal cell growth and totally absent in mammalian cells. The IPCs can also be mannosylated, forming MIPCs, which then can be further transformed into more complex forms, such as MIP_2_C, M_2_IPC, and possibly even more yet unknown forms. Studies on fungal content of these complex sphingolipids are hampered by the paucity of IPC lipid standards necessary for proper identification by liquid chromatography mass spectrometry (LC-MS).

Nonetheless, lipid analysis by LC-MS provided unprecedented information for understanding the role of these lipids on biological cellular functions, as limited information can be drawn by the level of expression of their corresponding genes/proteins. In fact, when in a pathway, the product of one reaction is used as a substrate of subsequent reactions, studying the analysis of the overall level of products and substrates overtime, rather than gene and protein expression, has provided more important insights on how the pathway responds to a stimulus or to a particular environment. These aspects have been exemplified using the biochemical systems biology and mathematical modeling approaches, which have allowed investigators to predict how the pathway responds upon various stimuli [25,26,27,28].

## 3. Molecules Targeting the Fungal Sphingolipid Pathway

There are two types of molecules targeting the fungal sphingolipid pathway: (i) synthetic drugs and (ii) molecules such as antibodies or antimicrobial peptides. Drugs inhibit the enzymatic activity of the enzymes involved in the biosynthesis or breakdown of sphingolipids. Molecules bind to specific sphingolipids, inhibiting their function.

## 4. Drugs

In the following sections, we discuss compounds that have been shown to directly inhibit the enzymatic activity of sphingolipid metabolizing enzymes. In fact, because of the high complexity of the pathway, it is expected that additional enzymes and proteins are indirectly involved in the sphingolipid synthesis. For instance, most of the sphingolipid enzymes are compartmentalized in specific organelles (e.g., ceramide synthases are located in the endoplasmic reticulum (ER), whereas glucosylceramide synthases 1 (Gcs1) and inositol phosphoryl ceramide synthase 1 (Ipc1) are located in the Golgi). That means that ceramide needs to be transported from the ER to the Golgi in order to be used by either Gcs1 or Ipc1. If this transport is blocked, IPCs or/and GlcCer synthesis will not occur. Therefore, a drug targeting the transport of vesicles containing ceramide from the ER to the Golgi (e.g., [N′-(3-bromo-4-hydroxybenzylidene)-2-methylbenzohydrazide (known as BHBM)) will significantly impact the synthesis of complex sphingolipids, even though the compound(s) does not directly inhibit any enzyme involved in the pathway illustrated in Figure 1 [29,30,31,32]. Similarly, compounds affecting fatty acid elongation, such as minimoidin, may also affect the synthesis of ceramide [33] because ceramide synthases are only able to incorporate specific fatty acids into DHS and PHS. Hence, the understanding of the compartmentalization of the sphingolipids within membranes, the compartmentalization of their metabolizing enzymes, and the transport of sphingolipids within the cell are all essential to understand how a specific enzyme regulates the expression and metabolism of any one sphingolipid. Whereas this knowledge is mostly available for mammalian sphingolipids, during the last few years, this knowledge has also become more widely available for fungal sphingolipids due to their increased interest in biomedical research.

## 5. Inhibitors of SPT

As mentioned, the serine palmitoyl transferase enzyme (SPT) catalyzes the condensation of serine and palmitoyl CoA to synthesize 3-ketodihydrosphingosine. This is an irreversible reaction and in fungi, the enzyme comprises of three subunits: Lcb1, Lcb2, and Tsc3. Tsc3 plays a major role in regulation of SPT activity by forming a heterotrimer with the Lcb1 and Lcb2 homologues. Interestingly, no mammalian homologue to Tsc3 has been identified in humans [34], even though the human enzyme also comprises of three subunits (SPTLC1, SPTLC2, and SPTLC3). However, whether inhibition of only Tsc3 would be sufficient to alter or block fungal SPT activity in pathogenic fungi awaits further studies. Although required for optimal SPT activity [35], deletion of Tsc3 in *S. cerevisiae* does not totally block SPT activity [36]. 

**Figure 2:**
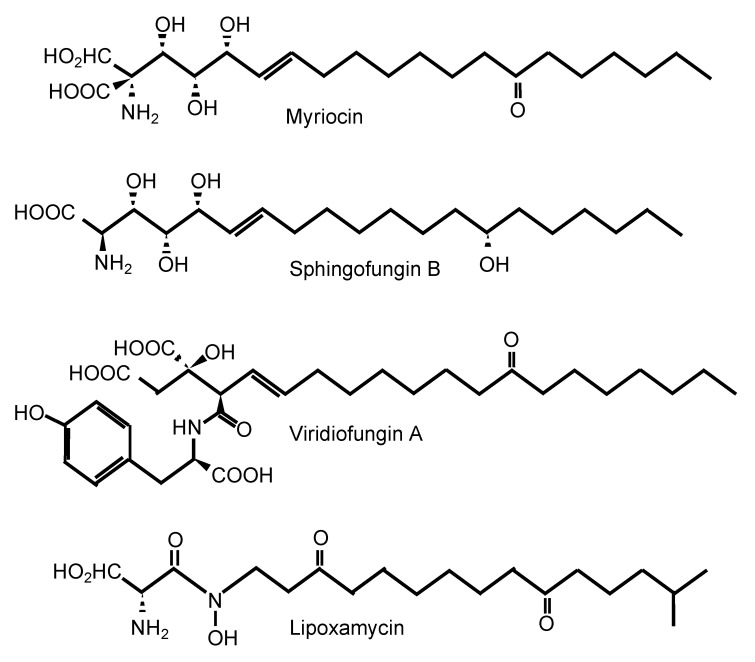
Inhibitors of serine palmitoyl transferase.

One of the most well-known SPT inhibitors is myriocin (Table 1 and Figure 2), also called ISP-I (for ImmunoSuPpressant from *Isaria*, although in several papers, it is also referred to as “ISP-1”). ISP-I was isolated from the fungus *Isaria sinclairii*, a vegetable used by the Chinese herbal medicine for “eternal youth” [37]. ISP-I was then found to have identical structure to myriocin, an antifungal agent isolated from the fungus *Myriococcum albomyces*, hence the name myriocin [38]. Myriocin is a potent immunosuppressant, and it inhibits both the fungal and mammalian SPT enzymes. Using kinetics, spectroscopy, and X-ray crystallography, the molecular mechanism of action of SPT inhibition by myriocin has been discovered using SPT from the bacterium *Sphingomonas paucimobilis* [34]. Myriocin forms an aldimine with pyridoxal-5′-phosphate at the active site of SPT, and the co-complex eventually degrades, acting as a suicidal inhibitor of SPT. Whether a similar mechanism is also present in the fungal and mammalian SPT enzyme awaits further studies. Solving the crystal structure of the fungal and/or mammalian SPT will allow a pinpointed target-drug design to identify a fungal-specific inhibitor. Of interest, the involvement of a third subunit (Tsc3) in the fungal SPT activity may hold promise as this subunit is absent in mammalian cells. In addition, the chemical structure of myriocin is similar to the structure of sphingofungins, viridiofungins, and lipoxamycin [39], which are all natural compounds isolated from *A. fumigatus*, *Streptomyces spp.*, and *Trichoderma viride*, respectively (Figure 2) [40,41,42,43]. Very interestingly, viridiofungins do not inhibit SPT of the common yeast *S. cerevisiae*, but they do inhibit SPT of the pathogenic fungus *C. albicans* [39]. This characteristic is unique to the viridiofungins, as other SPT inhibitors do similarly block SPT of *S. cerevisiae* and other fungi. However, this raises the possibility that if SPT specificity can be obtained between two yeast species, perhaps it could also be obtained between human and fungal SPT.

Lipoxamycin possesses antifungal activity and was discovered in the early 1970s [44], but it was not until 20 years later that its mechanism of action was elucidated and found to target SPT [43]. Unfortunately, it inhibits the mammalian enzyme 10-fold better than the fungal enzyme. In vitro experiments with lipoxamycin found the compound to be highly active against *C. neoformans* and *C. albicans* but not against *A. fumigatus*, but it was found to be highly toxic in mice when applied subcutaneously or topically.

Nonetheless, some myriocin derivatives may hold great promise for the treatment of fungal infections. For instance, simplifungin and valsafungins A and B block SPT activity and they do exhibit potent fungicidal activity against *C. albicans* [45]. The structure activity relationship of myriocin derivatives led to the discovery of totally new compounds, such as FTY720 and BAF312. Once phosphorylated in vivo, FTY720-P binds to its sphingosine-1-phophate receptors (S1Pr), which are internalized, making them unresponsive to the natural ligand sphingosine-1-phosphate (S1P). Thus, FTY720-P works as a functional antagonist on S1Pr. By blocking S1Pr1, FTY720-P prevents lymphocytes from exiting lymph nodes, resulting in dramatic lymphopenia, thus decreasing the blood–brain crossing of lymphocytes. This alleviates the pathological effects on the central nervous system during multiple sclerosis. BAF312 is a derivative of FTY720 and does not need to be phosphorylated for S1Pr binding. In addition, whereas FTY720 binds to S1Pr1, S1Pr3, S1Pr4, and S1Pr5, BAF312 binds to S1Pr1, S1Pr4, and S1Pr5, lacking its activity against S1Pr3.

These new immunosuppressant compounds, largely used to treat multiple sclerosis, lost their SPT inhibition but they did retain their antifungal activity. In fact, BAF312 was efficacious in improving mice survival when given to a primary infection of cryptococcosis [46]. FTY720 also exerted antifungal activity in vitro but not in vivo, when it is phosphorylated into FTY720-P [46], totally losing its antifungal effect. FTY720 was actually found to reactivate cryptococcosis from containment within the lung granuloma, a phenotype not shared by BAF312 [46].

The discovery of the SPT crystal structure [47] and the advent of *in silico* drug screening may have paved the way for exciting new tools when looking for new SPT inhibitors. These new opportunities may potentially help find new compounds with a broad spectrum of antifungal activity and mechanisms of action specifically targeting the fungal and not the mammalian SPT enzyme.

## 6. Inhibitors of Ceramide Synthases

Fumonisin and australifungin are the best known inhibitors of ceramide synthases (Table 1). Fumonisins are produced by *Fusarium* spp. and inhibit both fungal and mammalian ceramide synthases but have poor ability inhibiting fungal growth in vitro. In addition, fumonisin B1 is highly toxic to the liver and kidney from studies performed in animal models. On the other hand, australifungins produced by *Sporormiella australis* are highly active in vitro against *C. albicans*, *C. neoformans*, and *A. fumigatus*.

Blocking the synthesis of ceramide in yeasts is ideal because it will decrease the synthesis of essential complex sphingolipids (GlcCer or/and IPCs) and will increase the level of sphingoid bases (DHS and PHS), which are highly toxic because they act as detergents on membranes. In fact, studies of major fungi showed that ceramide synthases are important for fungal virulence, fungal growth in host environments, and fungal pathogenicity in animal models [48,49,50,51]. The key is to find specific fungal ceramide synthase inhibitors.

In recent years, two studies have supported the research and development of such inhibitors: one is a study reporting a new fluorescent assay for ceramide synthase activity (fungal or mammalian) in a 96-well plate, which will enable the screening of chemical libraries for more selective compounds, active against the fungal but not the mammalian ceramide synthases enzymes [52]. The other study described the discovery of the first specific inhibitor of the mammalian ceramide synthase 1 (CerS1) [53]. The authors chemically modified FTY720, which is a well known sphingosine analogue. However, the un-phosphorylated pro-drug is also able to inhibit the ceramide synthases as a collateral effect. Thus, this enabled the synthesis of new derivatives of FTY720 around the benzyl tail and found a specific human CerS1 inhibitor. This raises important questions about whether this or other derivatives would have any antifungal activity and whether a similar medicinal chemistry approach can be used to formulate a specific fungal Cer1 inhibitor. As mentioned, the un-phosphorylated FTY720 was indeed found to inhibit fungal growth. It will be exciting to study whether this antifungal activity is mediated though the inhibition of fungal Cer1 [53].

## 7. Inhibitors of Inositol Phosphorylceramide Synthase 1 (Ipc1)

Aureobasidin, khafrefungin, and rustimicin are the most known IPC inhibitors (Table 1). Ipc1 was in fact called Aur1, as the gene that gives resistance to aureobasidin A (AbA) [54]. AbA is a cyclic compound isolated from the fungus *Aureobasidium pullulans* [55], that potently inhibits Ipc1 almost exclusively in yeasts such as *C. albicans*, *S. cerevisiae*, and *C. neoformans*. Ipc1 in mold is less susceptible to AbA, and new AbA derivatives have been synthesized showing improved activity against *A. fumigatus* [56], but whether these compounds have similar selectivity to fungal compared to mammalian cells awaits further studies. Beside this, Ipc1 is an ideal fungal target because it is not present in mammalian cells and because Ipc1 is essential for fungal growth in yeasts. Thus, its inhibition will cause fungal cell death. Interestingly, AbA does not target the Ipc1 found in parasites, such as *Toxoplasma gondii*, even if it does inhibit the proliferation of the tachyzoite form of *Toxoplasma* [57]. This suggests that AbA may affect additional target(s) yet to be identified.

Similar to aureobasidins, pleofungins are nonadepsipeptides. Pleofungins were recently identified as novel inhibitors of Ipc1 isolated from the mycelial extract of the fungus *Phoma* spp. [58]. They inhibit Ipc1 of *A. fumigatus* more efficiently than the Ipc1 from *S. cerevisiae* and they show good fungal growth inhibition of *C. albicans*, *C. neoformans*, and *A. fumigatus*. These biological properties indicate that pleofungins belong to a novel class of IPC synthase inhibitors efficacious against both yeasts and molds [58]. Although expected not to be toxic to mammalian cells, the selectivity index of these compounds is largely unknown.

Other Ipc1 inhibitors include khafrefungin and rustimicin, both exhibiting good antifungal activity against *Candida* spp. and *C. neoformans* in vitro and in the animal models, but similarly to AbA, they have much less activity against *Aspergillus* spp.

Khafrefungin was first isolated from sterile fungal mycelia [33] and its inhibition of Ipc1 less attractive because it is time-dependent [59]. Rustimicin, also called galbonolide A, was isolated from *Micromonospora* spp. [60] and it is particularly active against plant-pathogenic fungi. Despite its non-ideal chemical properties (e.g., half-life less than 1h at pH 5 and below or 7 and above) [33], it is active against cryptococcosis in the animal model, although less effective than the standard of care [61].

The main challenges for the current Ipc1 compounds are to develop a simple, straightforward medicinal chemistry approach for their synthesis and structural modification for improving their spectrum of antifungal activity. A breakthrough came in recent years when small molecule inhibitors of plant Ipc1 were identified [62]. Because plant and fungal Ipc1 share a high degree of homology and because medicinal chemical modifications are much easier when using small molecules, this discovery may accelerate the research and development of novel Ipc1 inhibitors.

## 8. Targeting Sphingolipids Directly

Because fungal sphingolipids have different chemical structures compared to those of mammalian sphingolipids, they can be exploited as targets for antimicrobial peptides, such as defensins, to block their function. Several natural products have been identified to target GlcCer directly and modulate fungal growth. For instance, a defensin called *Raphanus sativus* antifungal protein 2 (RsAFP2) and heliomycin, are active against *C. albicans* and *Pichia pastoris* producing GlcCer, whereas strains lacking GlcCer are resistant. Interestingly, RsAFP2 and heliomycin do not interact with human GlcCer [63]. RsAFP2 also acts in other *Candida* non-*albicans* species producing GlcCer and, as expected, *C. glabrata* is resistant to RsAFP2 because this species does not make GlcCer [64]. Binding of RsAFP2 to GlcCer alters fungal cell wall shape and organization and, ultimately, affects the yeast–hyphae transition of *C. albicans*, an essential process for this fungus to cause disease.

In recent years, monoclonal antibodies against fungal GlcCer have been developed as fungal GlcCer is highly immunogenic. For instance, patients with cryptococcosis develop antibodies against GlcCer, and cell budding and cryptococcal growth in vitro are inhibited when treated with purified antibodies from these human sera [65]. The inhibition of cell budding was interesting [65], as later it was validated using a mutant lacking GlcCer, which also cannot bud [23], suggesting that GlcCer plays a key role in cytokinesis and that antibodies against GlcCer block this function. Monoclonal antibodies against fungal GlcCer were eventually produced and showed that passive administration of these antibodies protects mice from *C. neoformans* infection [66].

Blocking the function of GlcCer by these antibodies was also studied in other fungal systems and the results corroborated the studies performed in *C. neoformans.* For instance, treatment of *Fonsecaea pedrosoi* [67] and *Colletotrichum gloeosporioides* [68] with antibodies against GlcCer showed reduced fungal growth and conidia germination. Most interestingly, treatment with these antibodies enhanced the antifungal action of macrophages [67], suggesting a possible role in stimulating host cells against fungi. This phenotype was corroborated by studies in *Pseudallescheria*/*Scedosporium* complex, where antibodies against GlcCer were able to inhibit conidia germination and to enhance phagocytosis by macrophages, as well as being synergistic when combined with itraconazole [69].

In addition to GlcCer, certain forms of IPCs are also immunogenic. IgG2a monoclonal antibodies (named MEST-3) against fungal glycoinositol phosphorylceramide were produced and shown to strongly inhibit the differentiation and colony formation of *Paracoccidioides brasiliensis*, *Histoplasma capsulatum*, and *Sporothrix schenckii* [70]. Interestingly, the inhibitory effect observed with MEST-3 against IPCs was much stronger than the effect observed with the antibodies against GlcCer (MEST-2) [70]. Further studies are clearly needed in this area, but the results obtained so far clearly suggest that blocking sphingolipid function(s) using specific monoclonal antibody may hold great promise as a therapeutic option against invasive fungal infections.

## 9. Conclusions and Future Perspectives

Sphingolipids are critical players in fungal growth, replication, virulence, and pathogenicity and therefore can potentially serve as useful targets for the research and development of novel antifungal drugs. Cryptococcosis, candidiasis, and aspergillosis are severe invasive mycoses with high mortality in immunocompromised patients. The antifungal compounds for their standard of care are limited and fungal resistance is rapidly rising. There is an urgent need for antifungal compounds with novel mechanisms of action. The sphingolipid pathway offers several avenues as the fungal sphingolipids are structurally different than the mammalian counterparts or completely absent from the mammalian system altogether. Importantly, key fungal sphingolipid metabolizing enzymes are absent in mammalian cells or significantly different and have been shown to be targetable in the laboratory setting. One limitation is whether these compounds will be exclusively killing fungi and not mammalian cells. Toxicity studies and analysis of selectivity index will be able to address this limitation. As new technologies in drug design and drug screening are developed, the fungal sphingolipid pathway offers exciting opportunities for the development of antifungal compounds with a novel mechanism of action compared to current drugs and more selective to fungal cells resulting in less collateral effects to the host than current antifungals.

## Figures and Tables

**Table 1:** Inhibitors of the fungal sphingolipid biosynthetic pathway.

	Drug	Target	Pros	Cons
**Targeting sphingolipid enzymes**	SPT inhibitors: Myriocin Sphingofungin Viridofungin Lipoxamycin Simplifungin Valsafungins A&B	Serine palmitoyl transferase (SPT) (Sc Lcb1, Lcb2 and Tsc3) (Hu SPTLC1, SPTLC2, SPTLC3)	Highly active. Broad spectrum. Potential to improve selectivity toward the fungal homolog.	Highly toxic because they also inhibit human SPT1.
	Cer inhibitors: Australifungin Fumonisin B1	Ceramide synthases (Sc Lag1, Lac1, Lip1) (Hu CerS1, CerS2, CerS3, CerS4, CerS5 and CerS6)	Moderately active. High potential to improve selectivity and activity toward the fungal homologs.	Potential toxicity to mammalian cells.
	Ipc1 inhibitors: Aureobasidin A Khafrefungin Rustimicin Pleofungins	Inositol phosphoryl ceramide synthase 1 (Ipc1) (Sc Aur1) (Hu, absent)	Highly active. Limited toxicity to mammalian cells. High potential to improve broad spectrum.	Difficult to synthesize or/and to modify structure in order to improve activity.
**Target sphingolipids directly**	Defensins (RsAFP2)	Fungal GlcCer	Potentially active against all fungi producing GlcCer. Do not bind mammalian GlcCer.	Not active against fungi not producing GlcCer (e.g., *Candida glabrata*).
	Antibody against GlcCer	Fungal GlcCer	Potentially active against all fungi producing GlcCer. Synergistic when combined with antifungal compounds. Do not bind mammalian GlcCer.	Not active against fungi not producing GlcCer (e.g., *Candida glabrata*). Narrow spectrum of activity.
	Antibody against Glycoinositol phosphoryl ceramide	Fungal IPCs	Potentially active against all fungi producing IPCs. More effective than anti-GlcCer.	Not as active against fungi producing low level of IPCs (?).
